# A new fingerprint to predict nonribosomal peptides activity

**DOI:** 10.1007/s10822-012-9608-4

**Published:** 2012-09-29

**Authors:** Ammar Abdo, Ségolène Caboche, Valérie Leclère, Philippe Jacques, Maude Pupin

**Affiliations:** 1 LIFL UMR CNRS 8022 Université Lille 1 and INRIA Lille Nord Europe, 59655 Villeneuve d’Ascq cedex, France; 2 ProBioGEM, UPRES EA 1026, Polytech’Lille, Av P. Langevin, Univ Lille 1-Sciences et Technologies, 59655 Villeneuve d’Ascq cedex, France; 3 Computer Science Department, Hodeidah University, Al Hudaydah, Yemen

**Keywords:** Nonribosomal peptides, Target Prediction, Similarity searching, Drug discovery

## Abstract

Bacteria and fungi use a set of enzymes called nonribosomal peptide synthetases to provide a wide range of natural peptides displaying structural and biological diversity. So, nonribosomal peptides (NRPs) are the basis for some efficient drugs. While discovering new NRPs is very desirable, the process of identifying their biological activity to be used as drugs is a challenge. In this paper, we present a novel peptide fingerprint based on monomer composition (MCFP) of NRPs. MCFP is a novel method for obtaining a representative description of NRP structures from their monomer composition in fingerprint form. Experiments with Norine NRPs database and MCFP show high prediction accuracy (>93 %). Also a high recall rate (>82 %) is obtained when MCFP is used for screening NRPs database. From this study it appears that our fingerprint, built from monomer composition, allows an effective screening and prediction of biological activities of NRPs database.

## Introduction

For thousands years, natural products are an important source of drugs [1]. They are produced by marine or terrestrial organisms (plants, vertebrates, invertebrates…) and microorganisms (fungi, bacteria, algae). Many studies in the literature discuss the importance of natural products in drug discovery [2–5]. They are still important sources for many drugs in the market (e.g. morphine, cocaine, penicillin, taxols…) and are also good lead compounds suitable for further modification during drug development. Introducing a new compound on the market is time consuming and cost-intensive process [6, 7], in particular for natural products, so that strategies allowing time saving are welcomed.

The discovery of natural products requires specific steps as they are synthesized by living organisms. For example, scientists need to determine which organisms produce interesting compounds and define the conditions of production. The produced compounds have to be extracted from cultured media or from natural environments. Finally, chemical structures are determined. Those structures can, finally, be mimicked leading to artificial compounds. To reduce the time and cost of the specific steps, the optimal process is to predict the compounds produced by an organism directly from its genome sequence. This strategy can be particularly performed with nonribosomal peptides.

Those peptides are synthesized by a ribosome-independent cell machinery. This alternative pathway produces peptides using large multi-enzymatic complexes called nonribosomal synthetases (NRPSs) [8]. Those synthetases are composed of proteins organized in modules, each one being responsible for the incorporation of one specific amino acid in the final peptide. A relationship between specific signatures and a given incorporated amino acid have been determined from protein sequences of NRPSs [9–12]. So, from a genome sequence, bioinformatics analysis allows to extract genes coding for NRPSs, to deduce their protein sequences and to predict the amino acids incorporated in the produced peptide [13]. This predicted peptide can then be analyzed by bioinformatics tools to infer its putative activity.

We have collected nonribosomal peptides in Norine (http://bioinfo.lifl.fr/norine/) [14], the first and still unique computational resource dedicated to nonribosomal peptides (NRPs). Each peptide has a unique Norine identifier in the form NOR followed by a number of 5 digits. The database contains more than 1,100 nonribosomal peptides extracted from scientific literature with manually curated annotations such as biological activity, producing organisms or bibliographic references and, most importantly, their monomeric structure. We used the universal term monomer instead of amino acid because the entities encountered into those peptides do not only include the 21 proteogenic amino acids, but also derivates or unusual ones; other compounds such as carbohydrates or lipids can also be incorporated. Norine currently references 526 different monomers occurring in the listed peptides. The monomeric structures are encoded by undirected labelled graphs, with nodes representing monomers and edges corresponding to chemical bonds between them. One monomer can display more than two peptidic bonds, and non peptidic bonds are also observed in NRPs leading to peptides with cycles and/or branches. The database can be queried for peptide search through their annotations as well as through their monomeric structures. It also contains a section dedicated to the monomers incorporated into the peptides stored in Norine.

Due to the particular way of synthesis, nonribosomal peptides are a valuable source of a wide range of structural and biological activities, produced by microbial cells (typically bacteria and fungi). The NRPs may represent novel drugs for several pharmaceutical areas including antibiotics (penicillin and cephalosporin the precursor of which is ACV, NOR00006), antitumors (actinomycin D, NOR00228), and immunosuppressive agents (cyclosporin A, NOR00033). They can also be exploited in biotechnological applications such as biosurfactants. Their various and interesting biological activities almost comes from their original mode of synthesis that offers huge flexibility by including non proteogenic monomers and cycles and branching.

As they are small and exploited in pharmacology and biotechnology, nonribosomal peptides are usually represented by atomic structures and stored in chemical compounds databases. Classical chemo-informatics tools are applied to them as part of generalist chemical databases to predict their activity or do some structure search or comparison. Norine contains few links to structural conformation databases such as PDB (25 NRPs). However, the length of this data set is too low to be exploited for NRP comparison or activity prediction.

Due to the similar property principle, structurally similar compounds are expected to exhibit similar properties and similar biological activities. This principle is exploited for in silico drug discovery. The chemical compounds are virtually screened either by docking into the active site of interest or by virtue of their similarity to a known active. Many studies suggest that knowledge about a target obtained from known bioactive ligand is as valuable as knowledge of the target structures for identifying novel bioactive scaffolds through virtual screening [15, 16].

But, NRPs exhibit specificities in comparison to typical synthetic compounds (synthesis pathway, complex structures). So, published numerical representations for chemical compounds, such as fingerprints, may not be the optimal choice to represent NPRs. Our monomeric approach opens new ways to analyze them. As first observations showing that some monomers are specific to a given activity [17] were promising, we decided to further investigate the relationship between the NRP monomer structures and their activity.

In this paper a new fingerprint based on monomeric composition of NRPs is introduced. Monomer composition fingerprint (MCFP) is a new method for obtaining a representative description of NRP structures from their monomer composition in fingerprint form. In this work, we present experiments that show the usefulness of monomer composition fingerprint when used for similarity searching and activity prediction of NRPs.

## Materials and methods

### Monomer composition fingerprint (MCFP)

**Fig. 1:**
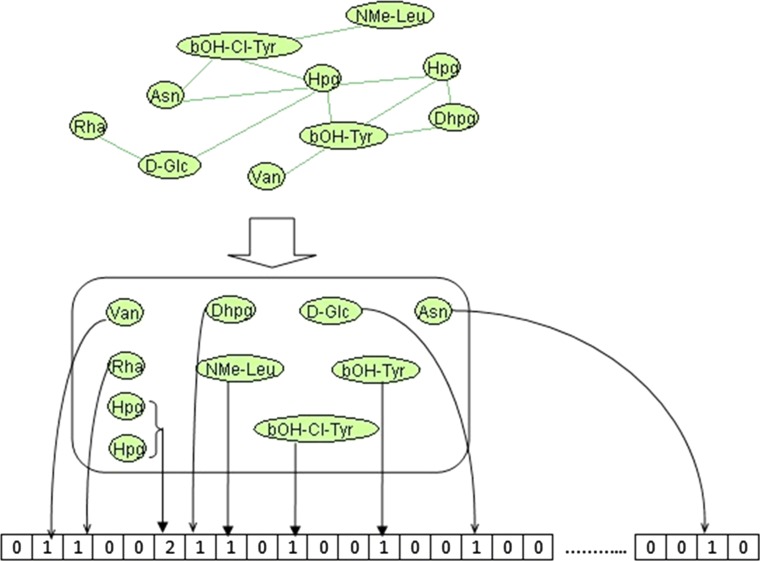
MCFP generation process

MCFP is represented as an integer vector, in which each element represents the presence (number of occurrences) or absence (“0” value) of a specific monomer. The process of generating the MCFP for each peptide starts by extracting the monomer compositions from Norine and then filling the corresponding positions in the MCFP vector. We use the 526 monomers referenced in Norine as individual elements (see Fig. 1). For example, the peptaibolin (NOR01028) is composed of the monomers NAc-Leu (N-acetyl-leucine), Aib (2-aminoisobutyric acid), Leu (leucine), Aib (2-Aminoisobutyric acid) and Pheol (phenylalaninol) and generates a fingerprint with three elements set to “1”, one to “2” and the rest (522) set to “0”. Four elements are “on” for this peptide of length five because the monomer Aib is repeated twice.

### Similarity search system

We use Tanimoto-based similarity search system (TAN). This system is based on the Tanimoto coefficient that is a well established method in similarity-based virtual screening and was therefore used as reference. In particular, the continuous form of the Tanimoto coefficient was used. If A_i_ and B_i_ represent the ith monomer occurrence in the peptides A and B, respectively, the similarity score S_A,B_ between peptides A and B was calculated by the following equation.

1 $$ S_{A,B} = {{\sum\limits_{i = 1}^{n} {A_{i} B_{i} } } \mathord{\left/ {\vphantom {{\sum\limits_{i = 1}^{n} {A_{i} B_{i} } } {\sum\limits_{i = 1}^{n} {(A_{i} )^{2} + \sum\limits_{i = 1}^{n} {(B_{i} )^{2} } - \sum\limits_{i = 1}^{n} {A_{i} B_{i} } } }}} \right. \kern-0pt} {\sum\limits_{i = 1}^{n} {(A_{i} )^{2} + \sum\limits_{i = 1}^{n} {(B_{i} )^{2} } - \sum\limits_{i = 1}^{n} {A_{i} B_{i} } } }} $$

The advantage of this score is the direct use of monomer occurrences in the equation and the neutrality of empty elements. This equation has been widely used for chemical similarity searching. However, a detailed study of fragment weighting schemes has recently suggested that superior screening performance is obtained if the square roots of the element occurrence frequencies are used rather than the unmodified frequencies [18–21]. We have hence carried out experiments in which the raw monomer occurrences in the TAN similarity measures are replaced by the square roots of those occurrences. The TAN coefficient varies between 0 (totally different monomer compositions) and 1 (identical monomer compositions).

### Activity prediction system

We use in our experiments three machine learning algorithms available in WEKA-Workbench [22, 23]. The naive Bayesian classifier [24], the linear (LibLinear) classifier [25], and the SMO classifier [26]. Details on these algorithms can be found in their references. The machine learning algorithms are used with their default settings in the WEKA-Workbench.

### Data sets

The data set for this study is taken from the Norine database (version of April 2012), which contains 1122 peptides with 11 distinct activities. We don’t consider the surfactant activity as it is more a physico-chemical property (being a lipopeptide or not) than a biological activity. The database is first filtered so that, activity classes containing less than 20 peptides are removed. Then, peptides with same monomer lists, even with different number of occurrences (same elements “on” in the MCFP), within an activity class are removed. Finally, we only consider the peptides with only one known activity. A total of 605 peptides were available for forming our test set, belonging to 5 different activity classes.

1. The **antibiotics class** (319 NRPs) includes different NRPs categories, which are peptaibols (linear peptides produced by fungi), glycopeptides (vancomycin-like with several cycles in their monomer structure), lipopeptides, pure peptides and even chromopeptides. It is to notice that, in Norine, 210 peptides share antibiotic with other activities (antitumor, toxin, surfactant or immuno-modulator). Those 210 peptides are included in the evaluation data set (see discussion section).
2. **Toxins** (157 NRPs) harbor different modes of action to kill cells. They are pure peptides or lipopeptides. In Norine, 103 NRPs that are toxins are also antibiotics, antitumors or surfactants. They are also in the evaluation data set.
3. **Siderophores** (82 NRPs) chelate (bind) iron molecules with specific monomers, including chromophores. They are mainly chromopeptides, but can also be lipopeptides or pure peptides. Among the 82 siderophore peptides, 18 are also known as surfactants.
4. **Antitumors** (25 NRPs) operate with different modes of action, being mainly pure peptides. In Norine, 71 NRPs that are antitumors are also antibiotics, toxins or immuno-modulator. They are also in the evaluation data set.
5. **Protease inhibitors** (22 NRPs) are all pure peptides. This class never crosses with other classes, as far as we know.

Performance of machine learning algorithms depends on the training data set (peptides with or without a given activity). The negative set, peptides without the studied activity, for any single activity class derives from the positive sets, that are peptides having any other activity.

### Validation

The similarity searching experiments were performed with 20 peptides selected randomly (as queries) from each activity class. The recall results were averaged over each such set of active peptides. The recall is the percentage of active peptides retrieved in the top-1 % or the top-5 % of the ranked list resulting from a similarity search.

For activity prediction experiments, 10-fold cross-validation was used to validate the results of different machine learning algorithms. In this cross-validation, the data set is split into 10 parts; one part is used for testing, the remaining 9 parts for training. This is repeated 10 times, so all the data have been used as test data once. Each activity class is tested against all the others, grouped. As in the case of many prediction methods, we used the F-measure as quality criterion to quantify the performance of MCFP with different classification algorithms. F-measure is defined as the harmonic mean of precision and recall. The precision is defined by *prec* = *tp*/(*tp* + *fp*) and the recall (or sensitivity) is defined by *rec* = *tp*/(*tp* + *fn*), where *tp*, *fp* and *fn* are the number of true positives, false positives, and false negatives, respectively. We also used accuracy (ac) and area under the Receiver Operating Characteristic (ROC) curve (AUC) measures to quantify the performance of MCFP with different classification models. Accuracy is the overall correctness of the model and is calculated as the sum of correct classifications divided by the total number of classifications *ac* = (*tp* + *tn*)/(*tp* + *tn* + *fp* + *fn*).

Further metrics of statistical performance analysis involved the ROC curve, which has been used in various fields (medicine, meteorology, etc.) [27] and also in drug discovery field [28]. A ROC curve describes the tradeoff between sensitivity and specificity, where the sensitivity is defined as the ability of the model to avoid false negatives, and the specificity relates to its ability to avoid false positives. The area under the ROC curve (AUC) is a measure of the model performance: the closer to 1, the better is the performance of the prediction.

## Results

### Similarity-based results

**Fig. 2:**
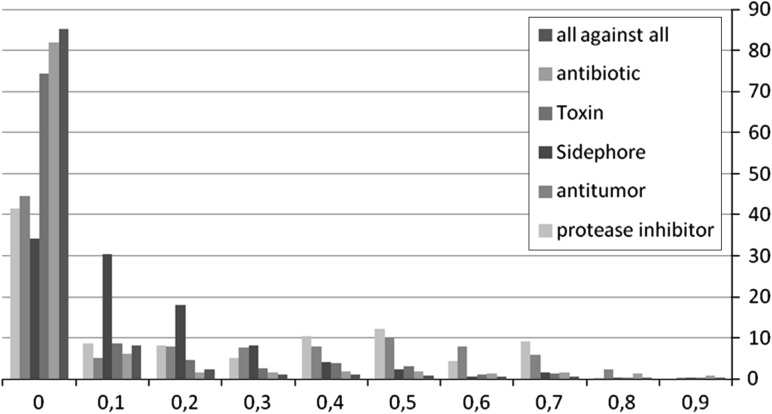
Histogram for pairwise similarity using Tanimoto coefficient

Details of the pairwise similarities among the activity classes are given in Table 1. A rough guide to the diversity of each of the chosen sets of NRPs is provided by matching each peptide with every other in its activity class (intra-class) or with all the 605 used in this study (inter-class), calculating the Tanimoto coefficient applied to MCFP. The class diversity is measured by computing the mean and the number of comparisons having a coefficient greater than or equal to 0.7 for these intra-class similarities. The histogram of Fig. 2 gives an overview of the pairwise distances obtained among intra- and inter-classes. The number of pairwise comparisons with a high score is low for all the classes, confirming a high diversity.

**Table 1:** Pairwise similarity and retrieval results for Tanimoto coefficient

Activity class	NRPs number	Pairwise TAN		TAN recall	
		Mean	% ≥0.7	Top 1 %	Top 5 %
Antibiotics	319	0.09	3.69	88.33	81.50
Toxin	157	0.09	1.65	75.00	59.33
Siderophore	82	0.18	2.11	100.00	90.83
Antitumor	25	0.27	8.67	67.50	45.21
Protease inhibitors	22	0.26	9.52	80.83	56.90
All against all	605	0.05	1.21		
Mean				82.33	66.75

The results for the searches in the data set are shown in Table 1. Each row corresponds to one activity class and lists the recall for the top 1 and 5 % of a sorted ranking when averaged over the ten searches for this activity class.

Results reported in Table 1 show that TAN system with MCFP obtains overall average recall rates of 82 and 67 % for top 1 and 5 %, respectively. It has the best performance for siderophore, antibiotics, and protease inhibitors activity classes while performing least well for antitumor and toxin. We observe a diminution of the recall between top-1 % and top-5 %.

### Biological activity prediction results

Visual inspection of the precision, recall, F-measure and accuracy rates in Table 2 enables one to make comparisons between the effectiveness of using MCFP with various prediction models. The MCFP with LibLinear approaches produce the best performance across the five activity classes, with SMO and NaiveB also performing well. In only one class (antitumor activity), the performance of the MCFP with different prediction models was low. In terms of the overall correctness of the prediction, MCFP fingerprint with different prediction approaches produced high accuracy rates, especially with LibLinear model (>93 %).

**Table 2:** Precision, recall, F-measure, accuracy and AUC rates for the prediction models

Activity class	Naïve Bayesian				LibLinear				SMO			
	Prec	Rec	F	AUC	Prec	Rec	F	AUC	Prec	Rec	F	AUC
Antibiotics	0.971	0.737	0.838	0.961	0.950	0.962	0.956	0.953	0.947	0.953	0.950	0.942
Toxin	0.656	0.898	0.758	0.946	0.899	0.904	0.902	0.934	0.889	0.917	0.902	0.937
Siderophore	0.890	0.988	0.936	0.998	0.988	0.963	0.975	0.981	1	0.951	0.975	0.994
Antitumor	0.471	0.640	0.542	0.935	0.696	0.64	0.667	0.814	0.696	0.640	0.667	0.868
Protease inhibitors	0.870	0.909	0.889	0.996	0.952	0.909	0.930	0.954	0.952	0.909	0.930	0.975
Accuracy	81.49				93.22				92.89			

In this study we used the ROC curve to study the performance of MCFP with different prediction models. Table 2 shows that the AUC value is always close to 1 (>0.93).

## Discussion

The main aim of this study is to introduce the monomer composition fingerprint as a useful representation for NRPs and then identify the effectiveness of using such representation in similarity-based and prediction of the activity for those peptides displaying many different biological activities. The best selection of descriptors/fingerprints is based on their accuracy in predicting the property/activity of a peptide from another peptide that is considered similar to it, by using either a similarity method, or a clustering or its k-nearest neighbors. For those descriptors, and for predicting the activity class of peptides, the best descriptors are those yielding the highest number of correct predictions (peptides with similar activity class), taking into account the total number of peptides having this activity in the database used. To achieve this aim, the Tanimoto similarity system (TAN, see Eq. 1) and three different machine learning approaches (NaiveB, LibLinear, and SMO) have been applied.

The TAN calculated on monomer composition fingerprint demonstrates good results for the recall computed on the top-1 %, except for the toxin and antitumor classes. The toxin class has only 14 % of specific monomers and shares up to 81 % of its monomers with the antibiotic class (see Table 4). So, they can match with antibiotics or other peptides because of their common monomers. This is not surprising as those activities are biologically closed and can even be both harbored by a single peptide (72 peptides of Norine are known to be antibiotics and toxins, we tested them as an evaluation data set). This is even worse for antitumors that have no specific monomers and share 96 % of their monomers with antibiotics and toxins. Their TAN recall is lower than the one of toxin. At the opposite, protease inhibitors have also no specific monomers and share 88 % of their monomers with antibiotics and toxins, but show the third best recall of the set. This is certainly due to the fact that they are small peptides (3, 4, 5 or 7 monomers) in comparison to the other peptides (mean number of monomers is around 10) and that their composition is specific of their activity. It is to notice that no peptide of Norine share protease inhibitor activity with another activity. Finally, antibiotics have the second best recall (88 %, in top 1 %), but it is not so good (as siderophore) because, as mentioned before, antibiotic class is constituted by several sub-groups that differ in monomer composition, structure and mode of action (they are peptaibols, glycopeptides, lipopeptides, pure peptides or chromopeptides). But the number of NRPs in each sub-class is sufficiently high to find similar peptides in top-1 % and top-5 % lists. Generally, the recall results presented here are highly interesting and promising. That is because this data set comprises heterogeneous activity classes which are normally considered as very challengeable in similarity-based searching. We plan to study more deeply the intra-class similarities to distinguish sub-classes among the actual activity classes, if some can be designed.

The prediction accuracy rates obtained with the three machine learning approaches are promising because they are higher than 90 %. Again, and for the same reasons, antitumor class gives lower rates. However, the mispredicted cases in antitumor class (see Table 3) are not really incorrect. This is because these cases are predicted as antibiotic and toxin classes and as we mentioned above, in Norine, NRPs that are antitumors can also be antibiotics and toxins. This finding is also supported by the number of common monomers between antitumor, antibiotic and toxin classes (Table 4). We plan to study the data sets within each class and across classes to improve the predictions. For example, isolated peptides can be removed from the classes.

**Table 3:** Confusion matrix for different prediction models

Activity class	Naïve Bayesian					LibLinear					SMO				
	a	b	c	d	e	a	b	c	d	e	a	b	c	d	e
a	235	64	9	11	0	307	9	0	3	0	304	11	0	4	0
b	5	141	1	7	3	9	142	1	4	1	9	144	0	3	1
c	0	1	81	0	0	1	2	79	0	0	2	2	78	0	0
d	2	7	0	16	0	6	3	0	16	0	4	5	0	16	0
e	0	2	0	0	20	0	2	0	0	20	2	0	0	0	20

a antibiotics, b Toxin, c Siderophore, d Antitumor, e Protease inhibitors

**Table 4:** Percentages of common and specific monomers

	Antibiotics (%)	Toxin (%)	Siderophore (%)	Protease inhibitors (%)	Antitumor (%)
Antibiotics	**38**	55	22	26	13
Toxin	81	**14**	24	20	39
Siderophore	74	55	**26**	32	13
Protease inhibitors	96	96	36	**0**	29
Antitumor	88	88	25	50	**0**

You should read the table by row. For example, antibiotics share 55 % of their monomers with toxins; antibiotics have 38 % of specific monomers. The sum of the rows is not equal to 100 % because some monomers are shared between several classes

The numbers in bold are the percentages of monomers specific of each activity

In order to assess the true predictivity of any model it is necessary to have an independent data set (evaluation data set) against which the model predictions can be compared. The evaluation data sets are different from the training data sets used to build the model. This approach makes it possible for users to judge the robustness and predictivity of the model when making predictions. Therefore, we predict the activity of 5 peptides that are not yet included in Norine (see Table 5) and built an exhaustive evaluation set with 232 peptides that are in Norine but not in the initial data set as they have at least two known activities. The data sets and predictions obtained with LibLinear method are presented in Tables 5 and 6. The correct activity, described in source papers, is predicted for 4 out of the 5 new peptides. Orfamide A is an antibiotic predicted as toxin, but crosses between antibiotic and toxin predictions are also observed in our initial data set. The results obtained for the evaluation set are promising as we predict correctly one of the activities for 83 % among the 237 tested peptides. This rate is similar to the one found with the cross-validation done with the initial data set, even if the activities represented in this set are challenging because they are the ones with the higher rate of crossing (antibiotic, antitumor and toxin). The prediction results for the evaluation data set clearly show the usefulness and robustness of our approach.

**Table 5:** Description and results for peptides that are not in Norine

Name	Ref.	Known activities	Predicted activity	Monomer composition
Coelichelin	[29]	Siderophore	**Siderophore**	D-Fo-OH-Orn, D-aThr, OH-Orn, D-Fo-OH-Orn
Hypomurocin A1	[30]	Antibiotic	**Antibiotic**	Ac-Aib, Gln, Val, Val, Aib, Pro, Leu, Leu, Aib, Pro, Leuol
Orfamide A	[31]	Antibiotic	Toxin	C14:0-OH(3); Leu,D-Glu, D-aThr, D-aIle, Leu,D-Ser, Leu, Leu, D-Ser, Val
Pyoverdin PSEN	[32]	Siderophore	**Siderophore**	ChrP, D-Ala, Asn,Dab, OH-His, Gly, Gly, Ser, Thr, D-Ser, OH-cOrn
TVB I	[33]	Antibiotic	**Antibiotic**	Ac-Aib, Gly, Ala, Val, Aib, Gln, Aib, Ala, Aib, Ser, Leu, Aib, Pro, Leu, Aib, Aib, Gln, Valol

The good predictions are in bold

**Table 6:** Results for evaluation data set extracted from Norine

NRPs number	Known activities	Predicted activity
62	Antibiotic, toxin	**32** **antibiotic**
		**29** **toxin**
		1 protease inhibitor
7	Antibiotic, toxin, surfactant	**7** **antibiotic**
5	Antibiotic, antitumor, toxin	**5 antibiotic**
17	Antibiotic, antitumor	**10** **antibiotic**
		6 toxin
		**1** **antitumor**
14	Antibiotic, antitumor, immunomodulating	8 toxin
		**6 antitumor**
95	Antibiotic, surfactant	**74** **antibiotic**
		5 toxin
		4 siderophore
		12 antitumor
29	Antitumor, toxin	2 antibiotic
		**22** **toxin**
		**5 antitumor**
3	Antitumor, immunomodulating	2 toxin
		**1 antitumor**

The good predictions are in bold

To improve the results in both similarity search and activity prediction, we will work on the fingerprints. On one hand, determining clusters of monomers will reduce the numbers of elements in the fingerprints and increase the common elements between peptides. On the other hand, adding of structure information such as monomer neighborhood will increase the number of elements in the fingerprints and improve the discrimination between two NRPs with similar monomer compositions but different structures.

The results obtained show that monomer composition fingerprint provides an interesting alternative to the widely used atomic fingerprints for similarity-based searching and biological activity prediction of nonribosomal peptides. However, beside the good performance of MCFP, it is efficient compared to any other representation approach, since dealing with fingerprint calculation is faster and conduct at minimal computational cost.

## Conclusion

In this paper, we present a new peptide fingerprint (MCFP) based on monomer composition of NRPs. Experiments with the Norine NRPs database, clearly show the usefulness and effectiveness of MCFP for similarity-based searching and biological activity prediction of nonribosomal peptides.
